# Genome-wide analysis of sulfotransferase genes and their responses to abiotic stresses in Chinese cabbage (*Brassica rapa L*.)

**DOI:** 10.1371/journal.pone.0221422

**Published:** 2019-08-19

**Authors:** Lu Jin, Ning Ouyang, Yong Huang, Chunlin Liu, Ying Ruan

**Affiliations:** 1 College of Bioscience and Biotechnology, Hunan Agricultural University, Changsha, China; 2 Key Laboratory of Crop Epigenetic Regulation and Development in Hunan Province, Changsha, China; 3 Key Laboratory of Plant Genetics and Molecular Biology of Education Department in Hunan Province, Changsha, China; 4 Agricultural College of Hunan Agricultural University, Changsha, China; Institute of Crop Science, CHINA

## Abstract

Sulfotransferases (SOTs; EC 2.8.2.-), which are widespread from prokaryotes to eukaryotes, constitute a multi-protein family that plays crucial roles in plant growth, development and stress adaptation. However, this family has not been systemically investigated in *Brassica rapa*. Here, a genome-wide systemic analysis of *SOT* genes in *B*. *rapa* subsp. *pekinensis*, a globally cultivated vegetable, were conducted. We identified 56 *SOT* genes from the whole *B*. *rapa* genome using Arabidopsis SOT sequences as queries and classified them into nine groups, rather than the eight groups of previous research. 56 *B*. *rapa SOT* genes (*BraSOTs*) were distributed on all 10 chromosomes except for chromosome 5. Of these, 27 *BraSOTs* were distributed in seven clusters on five chromosomes (ChrA01, ChrA02, Chr03, ChrA07, and Chr09). Among the BraSOT proteins, 48 had only one SOT_1 domain and 6 had two, while 2 had one SOT_3 domain. Additionally, 47 BraSOT proteins contained only known SOT domains. The remaining nine proteins, five in group-VIII and two in group-IX, contained additional transmembrane domains. Specific motif regions I and IV for 3′-phosphoadenosine 5′-phosphosulfate binding were found in 41 BraSOT proteins. Introns were present in only 18 *BraSOT* genes, and all seven *BraSOT* genes in groups VIII and IX had more than three introns. To identify crucial SOTs mediating the response to abiotic stress in *B*. *rapa*, expression changes in 56 *BraSOT* genes were determined by quantitative RT-PCR after drought, salinity, and ABA treatments, and some *BraSOT* genes were associated with NaCl, drought and ABA stress, e.g. *Bra017370*, *Bra009300*, *Bra027880*.

## Introduction

Sulfotransferases (SOTs) catalyze the transfer of a sulfonate group from 3′-phosphoadenosine 5′-phosphosulfate (PAPS) to an appropriate hydroxyl/amino group on numerous substrates [1]. This reaction, generally referred to as sulfation or sulfonation, has been found to occur in all organisms investigated to date [1] and is associated with various biological processes, such as cell communication, growth and development, and defense [2].

SOT substrates include numerous endogenous and xenobiotic molecules that contain hydroxyl or amino groups [3,4]. Sulfonation transforms drugs and other xenobiotic compounds into more water-soluble forms, thereby increasing their renal excretion and converting them into less toxic metabolites [5]; however, sulfonation can also activate molecules [6].

Because they all use PAPS as a co-substrate, SOT proteins are characterized by the presence of a histidine residue in the active site, defined PAPS-binding sites and a defined SOT domain (Pfam: PF00685) [1,7]. The PAPS-binding sites of SOTs are two highly conserved amino acid motifs known as regions I and IV [6,8,9], which are respectively localized near the N- and C-termini. SOT proteins in mammals were originally classified on the basis of their affinity for different classes of substrates. One group of SOT proteins, mainly membrane-associated, accepts macromolecules such as proteins/peptides and glycosaminoglycans [10] as substrates. The second group, which are typically soluble proteins, has substrates consisting of small organic molecules with diverse chemical structures, such as flavonoids, steroids and xenobiotics [2]. SOT proteins in plants can be divided into cytosolic and membrane-associated proteins. Thus far, most identified plant SOTs are cytosolic proteins with no transmembrane region and their exact localization is largely unknown. Only a few membrane-associated proteins, which are bound to the plasma membrane or localized in the Golgi apparatus, have been characterized in plants [11–13].

In *Arabidopsis thaliana*, 18 *SOT* genes (*AtSOT1*–*AtSOT18*) were initially identified on the basis of the sequence similarities of their translated products, with an average identity of 51.1%, and were classified into seven groups [14]. In 2008, another three *SOT* genes (*SOT19*-*SOT21*) were added to the *AtSOT* family to form group VIII [2]. Additionally, a tyrosylprotein SOT protein was identified, named AtTPST, that has low sequence similarity with other AtSOTs, possesses more amino acids, and lacks conserved amino acid sequences such as regions I to IV [13]. AtTPST is also the only Arabidopsis SOT protein containing a *Sulfotransfer_2* domain (PF03567) instead of a *Sulfotransfer_1* domain (PF00685) [1]. Arabidopsis SOTs have different substrate specificities; for example, the preferred substrate of AtSOT5 and AtSOT13 is flavonol [1,15,16], AtSOT8 functions as a flavonoid glycoside sulfotransferase [17], and AtSOT10 displays catalytic activity toward brassinosteroids [18], while AtSOT12 seems to have more functions with activities towards flavonone, brassinosteroids and salicylic acid and is involved in plant responses to salt, osmotic stress and hormone treatments [18–20]. AtSOTs can also sulfonate the bacterial-produced toxin cycloheximide [21].

In addition to genome-wide analyses of SOTs in *A*. *thaliana*, several limited studies have been conducted in other Brassicaceae species. Two SOT proteins, BNST3 and BNST4, have been characterized in *Brassica napus*. Comparative analysis of *B*. *napus* and *A*. *thaliana* databases has revealed at least 11 putative desulfoglucosinolate SOTs. In addition to the discovery of homologs of AtSOT16–18, phylogenetic analyses have revealed new subfamilies of desulfoglucosinolate SOTs not present in *A*. *thaliana* [22]. A comparative genomics study of *B*. *rapa* and *A*. *thaliana* identified all glucosinolate biosynthesis genes [23]. Twelve desulfoglucosinolate SOTs were identified and found to be differentially expressed in *B*. *rapa*, compared with only three present in Arabidopsis. In addition to Brassicaceae, SOTs have been identified in rice (*Oryza sativa* L.). In particular, the resistant allele of rice STV11 was found to encode an SOT (OsSOT1) catalyzing the sulfonation of salicylic acid [24].

Arabidopsis *SOT* genes are so far the most studied plant *SOT* genes. Given the wide range of functions and substrate specificities of SOTs and the very limited number of plant species in which they have been fully characterized, genome-wide studies of SOTs in more plant species will be useful to explore their functions in detail. Here, we identified 56 putative SOT-domain proteins in *B*. *rapa* across the whole genome. Phylogenetic analysis and classification was performed based on the *SOT* genes in *Arabidopsis*, followed by gene and protein structural characteristic analysis. Expression pattern of 56 *BraSOT* genes were also investigated via qRT-PCR. Our results provided basic resources for further functional research.

## Materials and methods

### Retrieval and identification of SOT domain proteins

Sequences of SOT domain proteins from *A*. *thaliana* were downloaded from the TAIR database (http://www.arabidopsis.org/) according to previous methods [2,13,14]. To identify all SOT proteins in *B*. *rapa*, all the AtSOTs were then used as queries for the blastp tool in the *Brassica* database (BRAD) (http://brassicadb.org/brad/index.php). An *E*-value of 10 based on the BLOSUM62 alignment score matrix was used as the matching criterion. Coding (CDS) and genomic sequences of BraSOTs were also verified according to CoGe FeatView (https://genomevolution.org/coge/FeatView.pl). The online Compute pI/Mw tool (http://web.expasy.org/compute_pi/) was used to calculate the theoretical isoelectric point and molecular weight of each protein [25].

### Chromosomal location and gene structural determinations

Location information for the *BraSOT* genes on the 10 *B*. *rapa* chromosomes, obtained from the latest version of BRAD, was depicted using the MapChart v2.3 (http://www.wageningenur.nl/en/show/Mapchart.htm) and Adobe Photoshop CS4 (http://www.adobe.com/) software. The online Gene Structure Display Server (http://gsds.cbi.pku.edu.cn) was used to generate IDD exon–intron structures including exon positions and gene length [26].

### Identification of conserved domains and phylogenetic analysis

The protein sequences obtained from BRAD were analyzed to determine their domain organization using the SMART sequence analysis tool and the Pfam database (http://smart.embl-heidelberg.de/). The domains were also verified and named according to the results of NCBI-CD searches against the CDD v3.14 database (47,363 position-specific scoring matrices). To visualize conserved motifs, SOT-domain amino acid sequences were analyzed with the WEBLOGO program (http://weblogo.threeplusone.com/). In addition, BraSOT protein motifs were identified using the MEME program (http://meme-suite.org/tools/meme), with motif discovery mode set to normal, motif number to 20, motif width to 5–50, and all other settings to default values. The results were manually adjusted.

Multiple sequence alignment of complete BraSOT and AtSOT sequences was performed using the T-Coffee program [27]. Phylogenetic and molecular evolutionary analyses were conducted in MEGA 6 [28]. Phylogenetic trees were constructed using the neighbor-joining method with the following parameters: gaps treated as missing data, the pairwise deletion option for total sequence analysis, the *p*-distance substitution method, and 1000 bootstrap replications to assess internal branch reliability [29].

### Plant materials and treatment

*B*. *rapa* seedlings were grown in liquid plant nutrient medium prepared with green-leaf-vegetable universal formula [30] under a 16/8h day/night photoperiod at 20±2°C and 50%–60% humidity. Two-week-old seedlings were prepared for stress treatment. For drought stress, seedlings were treated with 25% PEG-6000 once for 24 h. For salt stress, seedling roots were submerged in 200 mmol/L NaCl for 24 h. For ABA stress, plants were sprayed with 100 μmol/L ABA once and allowed to continue growing for 12 h. Plants grown in normal Hoagland nutrient solution were used as a control (S1 Fig). Drought and salinity-treated leaves were collected at 6, 12, 24 h after treatment. ABA-treated samples were collected at 3, 6, 12 h. Root, hypocotyl and cotyledon of the control seedlings were also collected. Samples were immediately frozen in liquid nitrogen and stored at −80°C for further analysis.

### Expression analysis by quantitative real-time PCR (qRT-PCR)

Total RNA was extracted using Trizol Reagent, according to the manufacturer’s specifications (Thermo Fisher, USA). First-strand cDNA was synthesized by reverse transcription of 2 μg of total RNA using the reverse transcription-PCR system (Thermo Scientific RevertAid RT Kit, USA).

To investigate the expression patterns of *BraSOT* genes under different stresses, all *BraSOT* genes were analyzed using real-time RT-PCR. The *Brassica* actin gene (Accession No. AF111812) was used as an internal control. Gene-specific primers (listed in S1 Table) were designed using the OligoArchitect Online software (http://www.oligoarchitect.com/OligoArchitect/).

The real-time PCR assay mix (20 μL) consisted of 3 μL cDNA sample (diluted 1:10), 10 μL 2× LightCycler® 480 SYBR Green I Master mix (Roche, USA), 0.5 μL of each primer (10 μM) (S1 Table), and 6.0 μL distilled deionized H_2_O. Each real-time PCR assay was run on a LightCycler® 480 System (Roche, USA) with an initial denaturation at 95°C for 6 min, followed by 45 cycles of 95°C for 15 s and 60°C for 30 s. The resulting fragments were subjected to melting-curve analysis to verify the presence of gene-specific PCR products. The melting-curve analysis was performed directly after real-time PCR under conditions of 95°C for 5 s followed by a constant increase from 65 to 97°C at a 2.50°C/s ramp rate. The 2^−ΔΔCt^ method was used to calculate the relative amount of template present in each PCR amplification mixture [31].

## Results

### Identification of SOT proteins in *B*. *rapa*

Using the approaches described in the Methods to identify all the SOT-domain containing proteins in *B*. *rapa*, we obtained 56 proteins. To confirm these 56 putative SOT proteins, we performed SMART analysis and the results showed that all 56 proteins contained a sulfotransferase domain according the Pfam database. Therefore, we identified 56 BraSOT proteins in *B*. *rapa*, compared with 22 proteins in Arabidopsis. The BraSOT amino acid lengths ranged from 65 (Bra017006) to 589 (Bra017364) residues. Excluding the two shortest BraSOT proteins (Bra036654 and Bra017006), the average BraSOT length was 325.4 amino acids (Table 1), similar to the AtSOTs in Arabidopsis.

**Table 1:** Features of the SOT domain protein family in *Brassica rapa*.

Gene ID(Brassica Database)	Gene length(bp)	ORF length(bp)	Protein length(aa)	No.of introns	Mol.wt (kDa)	pI	Chromosome
Bra005879	1773	948	315	1	36.48	5.95	3
Bra005876	981	981	326	0	37.25	5.60	3
Bra005878	977	978	325	0	37.24	5.91	3
Bra009164	994	918	305	1	34.65	6.40	10
Bra004046	981	981	326	0	37.76	5.61	7
Bra016726	981	981	326	0	37.33	5.83	8
Bra004349	984	984	327	0	37.75	5.65	7
Bra036654	279	279	92	0	10.76	4.66	9
Bra034520	1350	852	283	1	32.57	9.47	Scaffold000096
Bra034521	5145	1752	583	3	66.08	6.01	Scaffold000096
Bra027666	879	879	292	0	34.08	6.27	9
Bra017006	198	198	65	0	7.57	6.05	4
Bra026450	990	990	329	0	37.98	5.97	1
Bra041015	990	990	329	0	38.14	5.71	Scaffold000379
Bra010899	978	978	325	0	37.64	5.45	8
Bra034065	963	963	320	0	36.94	6.56	1
Bra034066	990	990	329	0	37.57	5.59	1
Bra026540	957	957	318	0	36.96	6.42	2
Bra027963	1011	1011	336	0	38.82	6.02	9
Bra036913	1008	1008	335	0	38.76	5.76	1
Bra017371	824	720	239	1	26.83	5.19	9
Bra017369	768	768	255	0	28.58	5.13	9
Bra017368	972	972	323	0	36.62	6.04	9
Bra017365	983	912	303	1	34.45	6.11	9
Bra017373	807	807	268	0	30.25	6.36	9
Bra017364	3312	1770	589	7	66.92	6.03	9
Bra017370	987	987	328	0	37.17	6.47	9
Bra017372	742	638	211	1	23.94	5.55	9
Bra026535	1050	1050	349	0	39.41	5.82	2
Bra026538	483	387	128	1	14.35	5.06	2
Bra026539	780	780	259	0	29.61	6.83	2
Bra026536	567	567	188	0	21.30	8.64	2
Bra005921	1083	1083	360	0	41.19	6.48	3
Bra009300	1056	1056	351	0	40.48	5.81	10
Bra028711	1095	1095	364	0	41.56	7.65	2
Bra003818	1035	1035	344	0	40.14	5.50	7
Bra003726	995	873	290	1	33.59	5.69	7
Bra003817	1047	972	323	1	37.20	5.77	7
Bra027623	1059	1059	352	0	40.54	6.33	9
Bra003819	390	390	129	0	14.95	5.56	7
Bra027118	1107	1107	368	0	42.47	5.61	9
Bra027880	1020	1020	339	0	39.37	5.80	9
Bra027117	1065	1065	354	0	40.68	5.60	9
Bra031476	951	951	316	0	36.20	5.13	1
Bra015938	1035	1035	344	0	40.14	5.08	7
Bra015936	1041	1041	346	0	40.18	5.87	7
Bra025668	1014	1014	337	0	38.90	5.48	6
Bra008132	1020	1020	339	0	39.25	5.50	2
Bra015935	561	561	186	0	21.60	5.03	7
Bra036094	1399	990	329	4	37.93	9.27	9
Bra012895	1140	927	308	3	35.43	8.74	3
Bra011532	1762	1032	343	4	39.85	8.83	1
Bra013096	1958	1035	344	5	39.85	9.13	3
Bra015232	2089	1035	344	5	39.77	9.07	7
Bra018664	3134	1497	498	11	57.03	9.40	6
Bra030710	2869	1491	496	11	57.06	9.10	8

Abbreviations: ID, identification; bp, base pair; aa, amino acids; pI, isoelectric point.

### Chromosomal localization of the *BraSOT* gene family

**Fig 1:**
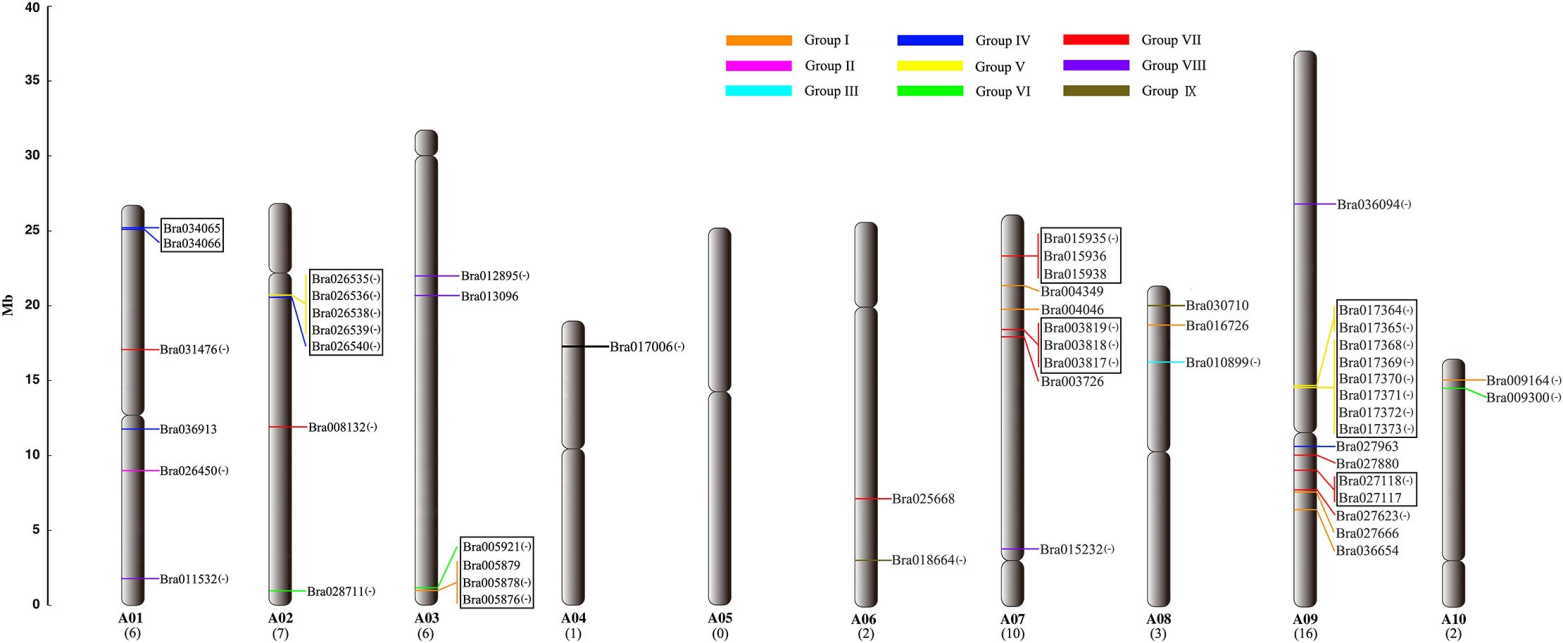
Distribution of 53 sulfotransferase (*SOT*) genes on the 10 *Brassica rapa* chromosomes. The positions of each gene are based on information in the most recent version of the *Brassica* database. Three *BraSOT* genes on the scaffold (Bra034521, Bra041015 and Bra034520) could not be anchored to a specific chromosome. The number under each chromosome code is the number of genes on that chromosome. The group (I–IX) corresponding to each *BraSOT* gene on a chromosome is indicated by a colored line. Boxes indicate gene clusters.

Based on the latest information available in BRAD, we localized 53 of the 56 genes encoding SOT proteins on all 10 *B*. *rapa* chromosomes except for chromosome A05 (Fig 1). The locations of three *BraSOT* genes (*Bra034520*, *Bra034521*, *Bra041015*) assigned to scaffolds could not be determined. As shown in Fig 1, the *BraSOT* genes were unevenly distributed on the *B*. *rapa* chromosomes. Specifically, there were no *SOT* genes on chromosome A05, 1 on A04, 2 on A06 and A10, 3 on A08, 6 on A01 and A03, 7 on A02, 10 on A07 and 16 on A09. As defined by Holub et al. (2001) [32], a chromosome region containing two or more genes within 200 kb is considered to be a gene cluster. In *B*. *rapa*, 27 *BraSOT* genes formed seven such clusters (Fig 1). In one case, eight (*Bra017364*, *Bra017365*, *Bra017368*, *Bra017369*, *Bra017370*, *Bra017371*, *Bra017372* and *Bra017373*) of the 16 *BraSOT* genes on chromosome A09 were closely spaced together in a tandem fashion within a 120-kb region. The seven identified clusters were distributed as follows: two each on chromosomes A07 and A09 and one cluster each on chromosomes A01, A02 and A03. No clusters were located on chromosomes A04, A06, A08 or A10.

### Phylogenetic analysis of the SOT family in *B*. *rapa*

**Fig 2:**
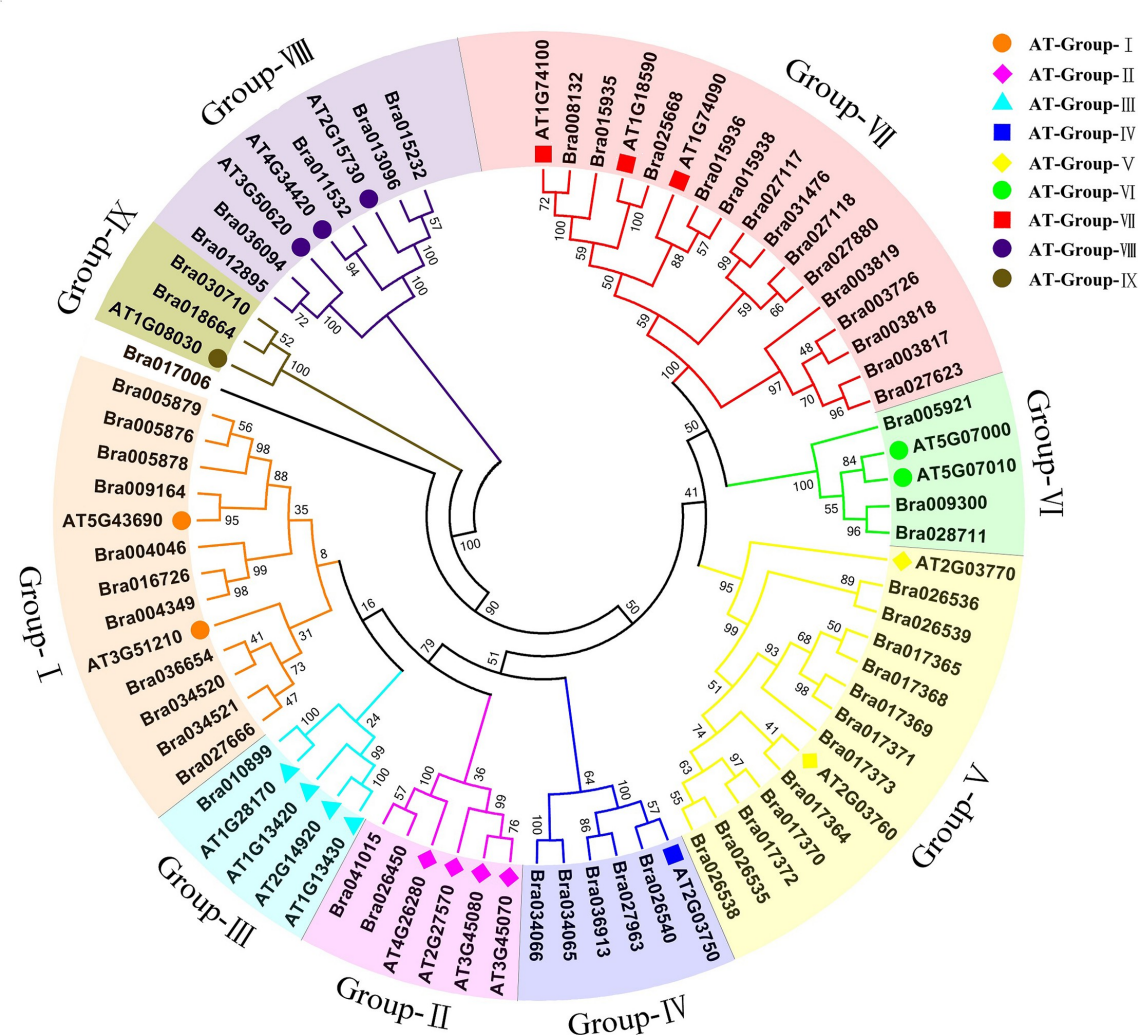
Phylogenetic tree of sulfotransferase (SOT)-domain proteins in *Arabidopsis thaliana* and *Brassica rapa*. The tree includes 56 BraSOT and 22 AtSOT proteins. Values above and below branches are bootstrap percentages based on 1000 replicates. AtSOT proteins are indicated by colored symbols corresponding to SOT groups I to IX.

To reveal the phylogenetic relationships among all 56 BraSOT proteins, phylogenetic analysis was performed with the MEGA software using the full-length amino acid sequences of the SOT proteins and a bootstrap value of 1000 (Fig 2). To obtain a reliable classification within different subfamilies, the SOT domain proteins from *Arabidopsis* were used as references. Similar to Arabidopsis, the results showed that the all but one BraSOT protein could be divided into nine subfamilies according to their sequence similarities. One protein with a very short amino acid sequence (Bra017006) formed a single branch. Considering this protein was too short to constitute a complete SOT domain, we did not classify it into any group. The classification of AtSOT proteins on this phylogenetic tree was identical to that in previous research [2], while the proportions of each group in the whole set of SOT proteins in *B*. *rapa* and Arabidopsis were different.

### Identification of conserved SOT domains and motifs in the BraSOTs

**Fig 3:**
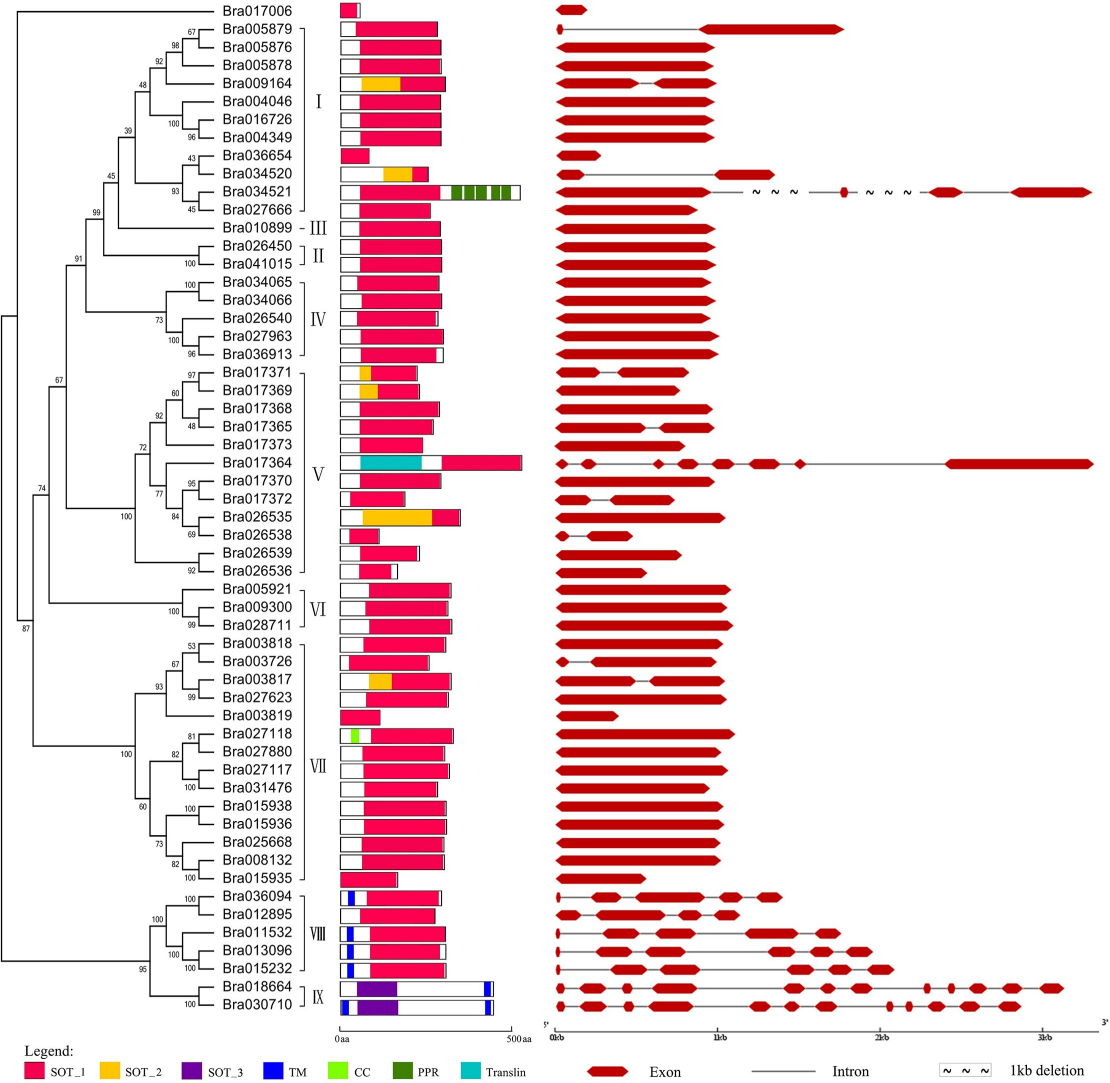
Conserved domains and gene structure of the sulfotransferase (SOT) family in *Brassica rapa*. Left panel: Neighbor-joining tree showing phylogenetic relationships of 56 BraSOT protein family members. Roman numerals correspond to the nine recognized groups of *SOT* genes. Numbers above and below branches are bootstrap percentages. Middle panel: Conserved domains in BraSOTs. The SOT_1 domain represented by a yellow bar is a second copy of the SOT_1 domain. Abbreviations: TM, transmembrane; CC, coiled-coil. Right panel: Exon–intron structure of *BraSOT* genes drawn with GSDS v2.0. Red bars and lines represent exons and introns, respectively.

Three important SOT-related Pfam domains, *Sulfotransfer_1* (PF00685, SOT_1), *Sulfotransfer_2* (PF03567, SOT_2) and *Sulfotransfer_3* (PF13469, SOT_3) with average lengths of 230.1, 218.3 and 224 amino acids, respectively, were identified in the BraSOTs. To analyze the three SOT domains and other recognized conserved domains in the full-length BraSOT amino acid sequences, a domain location map was constructed according to the domain information obtained from the SMART database [33] (Fig 3). Most of the BraSOT proteins contained a SOT_1 domain, and only Bra018664 and Bra070710 contained a SOT_2 domain. Six BraSOT proteins in groups I, V and VII possessed two tandem SOT domains. The SOT domain was generally near the C-terminus in most of the BraSOT proteins, while the SOT domain of three proteins (Bra034521, Bra018664 and Bra030710) was near the N-terminus. Only nine BraSOTs contained additional conserved domains. In particular, six BraSOTs in groups VIII and IX contained a transmembrane domain (TM). The Group-I protein Bra034521 contained several tandem pentatricopeptide repeat domains next to the C-terminus following the SOT domain. Bra017364 in Group V contained a translin domain, while Bra027118 in Group VII possessed a coiled-coil domain.

**Fig 4:**
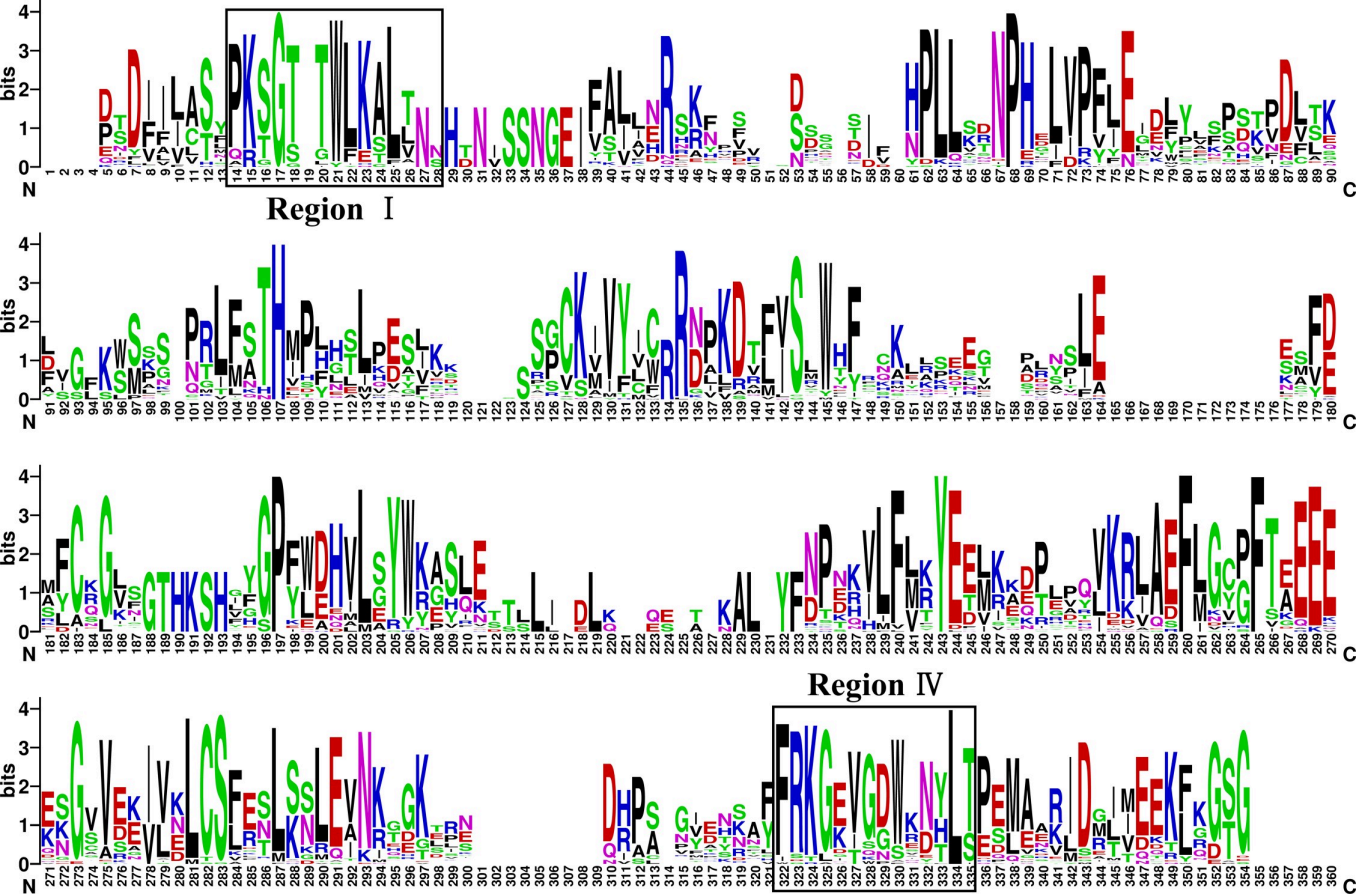
The SOT domain is highly conserved in many regions in *Brassica rapa*. In this figure, the height of each amino acid character represents the relative conservation of the sequence at that position. Highly conserved regions I and IV are indicated with boxes.

**Fig 5:**
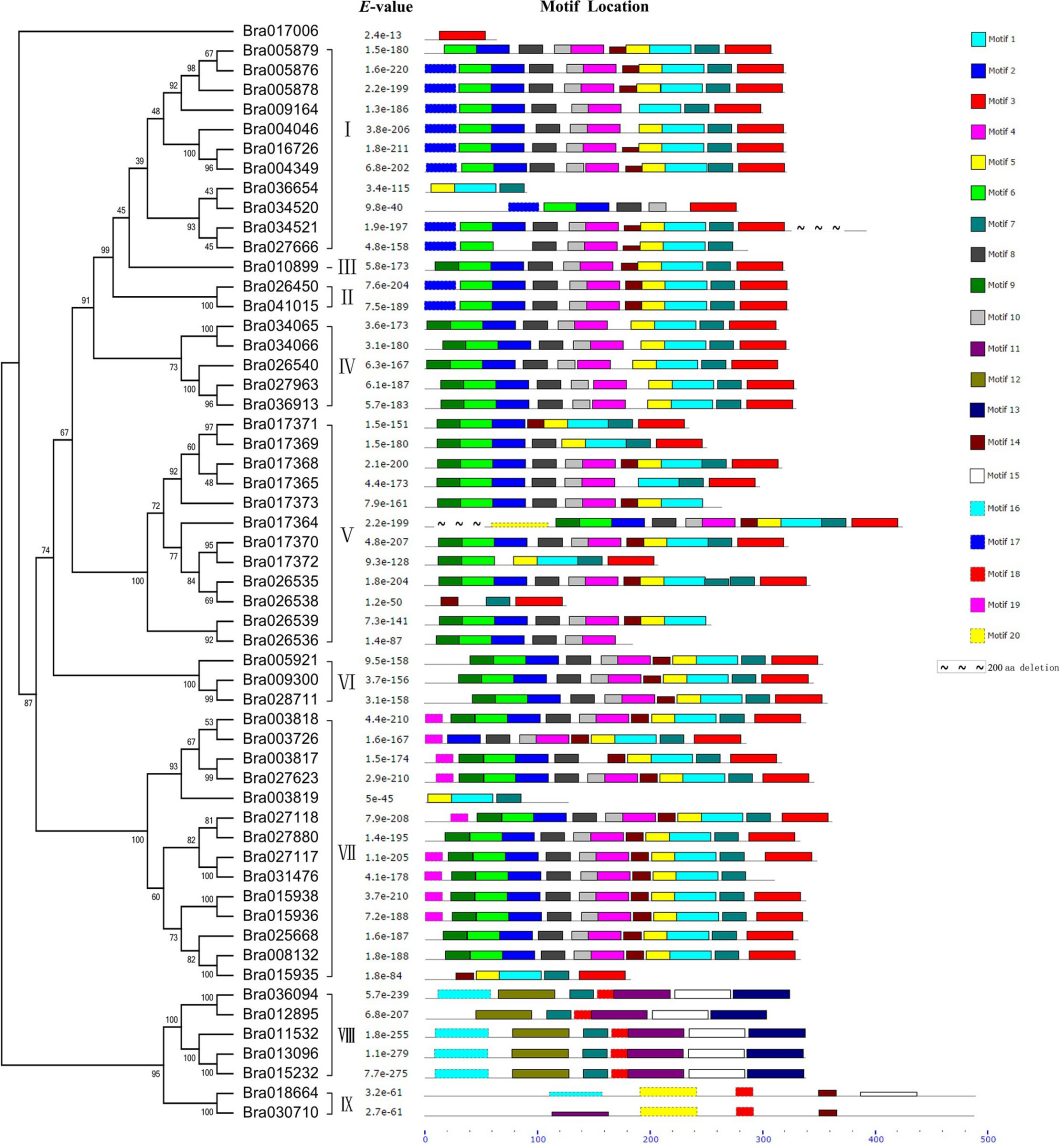
Conserved motifs of BraSOT proteins. Phylogenetic relationships, SOT groups, E-values, and locations of motifs in BraSOT domain sequences are shown. Values above and below branches in the tree are bootstrap percentages. Motifs are represented by the colors shown in the legend on the right. Motifs 2 and 3 include regions I and region IV, respectively. The height of a block indicates the significance of the match, as taller blocks are more significant.

According to previous research, all SOT proteins have conserved amino acid regions, designated regions I and IV, that are involved in PAPS binding [6,8];. The BraSOT proteins contained both regions, and their consensus sequences were highly similar to those of *A*. *thaliana* (Fig 4). Region I is located near the N-terminus, whereas region IV is adjacent to the C-terminus. To visualize the distribution of highly conserved motifs in the BraSOT proteins, 20 specific conserved motifs were identified using the MEME motif search tool. The distribution of these motifs in BraSOT proteins is displayed in Fig 5.

Among the 56 BraSOT proteins, eight motifs appeared at least 40 times and nine were present fewer than 12 times. The most frequent motif (50 times) was Motif 7. None of these motifs were present in all BraSOT proteins. The motif types of groups VIII and IX appeared to be quite different from those of the other BraSOT groups. Motifs 11, 12, 13, 15, 16 and 18 were specific to groups VIII and IX. Motifs 2 and 3 respectively contained region I at the C-terminus and region IV near the N-terminus, the two amino acid regions involved in PAPS binding. Of the 56 BraSOT proteins, 41 possessed both regions I and IV, while four only contained region I and another four only contained region IV.

### Gene structure analysis of the *BraSOT* gene family

A map of exon–intron structure based on the *B*. *rapa* genome and *BraSOT* CDS sequences was generated with the GSDS tool [26] to reveal the gene structures (Fig 3). This map revealed that 32.14% of *BraSOT* genes contained introns, a level similar to that of *A*. *thaliana SOT* genes (31.82%). Among the 18 intron-containing *BraSOT* genes, 9 genes had 1 intron, 2 had 3 introns, 2 had 4 introns, 2 had 5 introns, 1 had 7 introns and 2 genes from group IX had 11. Seven of the nine *BraSOT* genes containing more than three introns were members of groups VIII and I; the other two genes were in groups I and V.

### Expression profiles of *BraSOT* genes in different tissus and the response to drought, salt and ABA

**Fig 6:**
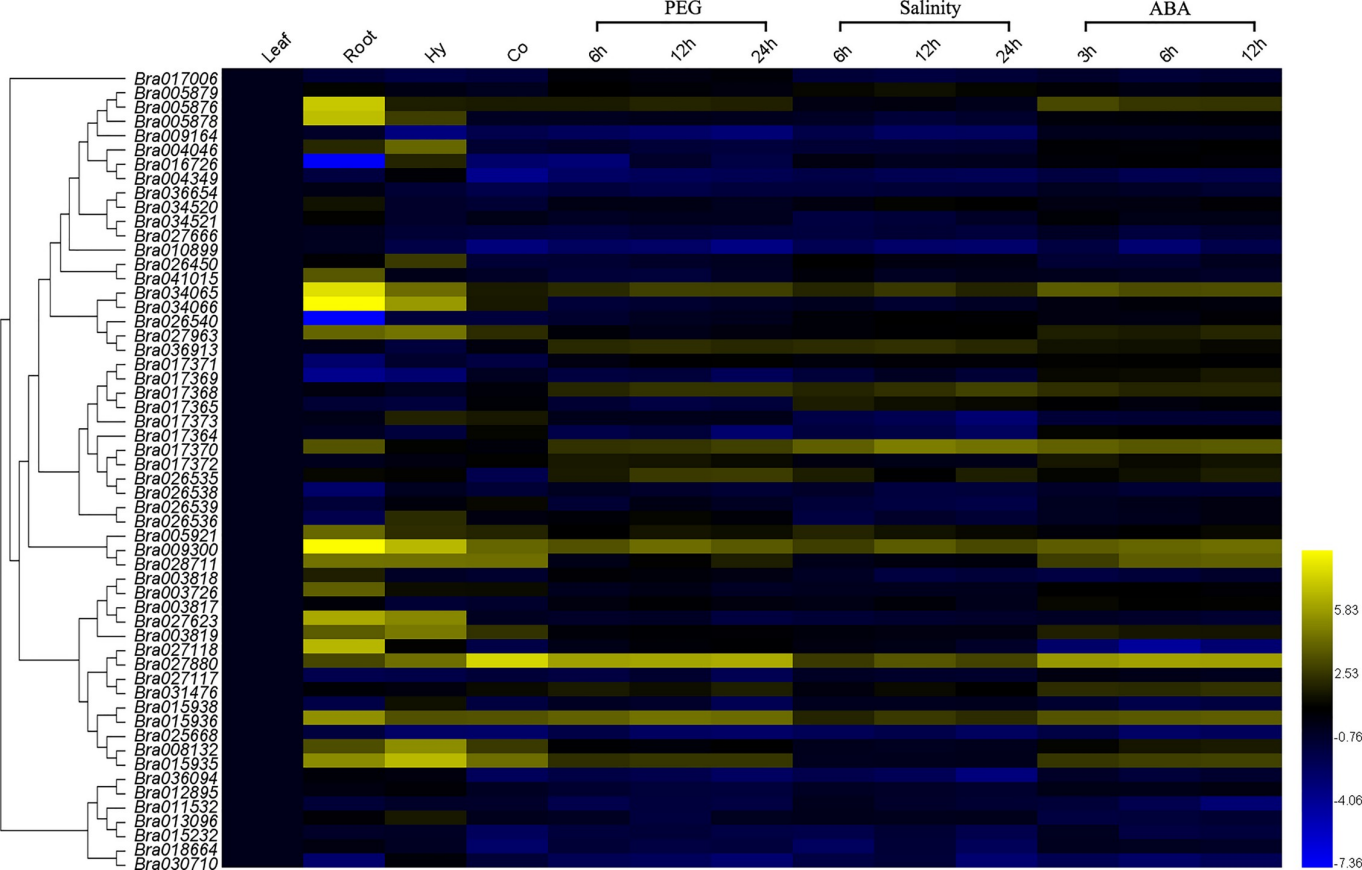
Expression modes of 56 *BraSOT* genes in brassica tissues and treated with 25% PEG-6000, 200mmol/L NaCl and 100μmol/L ABA. The bars display the relative gene expression levels, calculated based on the 2^−ΔΔCt^ method. Gene expression level in leaf with no treatment was used as the reference for calculation. Thus different colour present horizontal comparison of relative expression. The expression level is equal to the mean values and transform log_2_ values. Hy, hypocotyl; Co, cotyledon; polyethylene glycol (PEG), drought stress; Salinity, salt stress; ABA, abscisic acid treatment.

To investigate the potential functions of BraSOT family, Real-time RT-PCR was used to analyze the expression of *BraSOT* genes in different tissues of *B*. *rapa* seedlings. The gene expression level in leaf with no treatment was used as the reference for calculation, and results are presented in S2 Table. *BraSOT* genes showed different expression patterns in different tissues (Fig 6).Notably, 3 genes in group IV (*Bra034065*, *Bra034066*, *Bra027963*), all in group VI (*Bra005921*, *Bra009300*, *Bra028711*) and most in group VII showed higher transcription in root, hypocotyl and cotyledon than in leaf (Fig 6). The tissue-specific expression *BraSOT* genes indicate the probable roles of members of different groups in various physiological and developmental processes.

To further explore *BraSOT* gene expression patterns under abiotic stresses and identify genes important for improving tolerance to abiotic stresses, *B*. *rapa* seedlings were challenged with 25% PEG-6000, 200mmol/L NaCl and 100μmol/L abscisic acid (ABA). The expression patterns of most *BraSOT* genes showed transcriptional changed under drought, salinity, and ABA stresses in different treatment time points, and this suggested that the response of *BraSOTs* to multiple stresses is a dynamic process (S2 Table and Fig 6). For drought treatment, 15 *BraSOTs* were up-regulated at all of the time points. The expression levels of *Bra026536*, *Bra005921* and *Bra028711* increased at one or two time points. While 28 tended to be down-regulated in response to drought treatment, speciFIcally those belongs to group I, group II, group VIII and group IX. The change of the other 13 *BraSOTs* expression levels is not signiFIcantly at all time points under drought stress. Under salinity stress, the expression levels of *Bra034065*, *Bra036913*, *Bra017368*, *Bra017370*, *Bra009300*, *Bra027880* and *Bra015936* at three-time points considerably up-regulated. Similar to drought response, most *BraSOT* genes expression in group I, group II, group VIII and group IX decreased after salt treatment. For ABA teatment, the expression level of *Bra005876*, *Bra034065*, *Bra017370*, *Bra009300*, *Bra028711*, *Bra027880*, *Bra015936* and *Bra015935* were significantly increased. 15 *BraSOTs* of 56 tended to be down-regulated. Taken together, we found the expression patterns of some *BraSOT* genes are similar under drought, salinity, and ABA treatments (Fig 6).

## Discussion

Members of the SOT family have been found in most organisms including mammals and plants. These enzymes use PAPS as the sulfuryl donor and transfer the sulfonate group to an appropriate hydroxyl group on several classes of substrates. Although several SOTs have been identified and characterized in mammals and plants, the biological functions of SOTs in plants are still largely unknown. SOTs modulate physiological processes such as growth, development, and adaptation to stress by affecting the biological activity of certain compounds [11,18,21]. In *Brassica napus*, a SOT catalyzes the O-sulfonation of brassinosteroids and thereby specifically abolishes the biological activity of 24-epibrassinolide [14]. To further determine the roles of this multi-protein family, identification and analysis of more SOTs in plants is necessary.

### Identification and classification of *B*. *rapa* SOT proteins

In the present study, we analyzed the SOT protein family in *B*. *rapa* and identified 56 BraSOT proteins. The density (number/genome size) of *SOT* genes in the *B*. *rapa* genome is 0.197, similar to that in Arabidopsis (0.163) and much higher than the value of 0.093 in *O*. *sativa* [34]. The 56 SOT proteins have an average length of approximately 325.4 amino acids, similar to SOTs in *A*. *thaliana*. Fifty-three of the 56 *BraSOT* genes were localized to the 10 chromosomes of *B*. *rapa* and were found on all chromosomes except chromosome 5 (Fig 1). Because only scaffold information was available for the other three *BraSOT* genes (Table 1), they could not be positioned on an exact chromosome. Seven gene clusters including 27 *BraSOT* genes were detected on five chromosomes and accounted for nearly half (48.21%) of all *BraSOTs*. For example, eight *(Bra017364*, *Bra017365*, *Bra017368*, *Bra017369*, *Bra017370*, *Bra017371*, *Bra017372* and *Bra017373*) of the 16 *BraSOT* genes on chromosome A09 were tandemly clustered in a 120-kb region. The distribution of gene clusters on A02, A03, A07 and A09 suggests that tandem duplication has occurred during the process of *Brassica* evolution [35].

Considering the results of other studies, the division of the 56 *BraSOT* genes into nine groups is reasonable. In Arabidopsis, 18 *SOT* genes were initially classified into seven subfamilies (I–VII) [14]. In 2008, three new *AtSOT* genes (*At3G50620*, *At2g15730* and *At4g34420*) were added to the *AtSOT* gene family and were respectively designated *AtSOT19*, *AtSOT20* and *AtSOT21*. These three new genes were classified into group VIIIof the Arabidopsis *SOT* gene family [2]. A tyrosylprotein *SOT* gene (*AT1G08030*) was identified in 2009 and named *AtTPST* [13]. Because the AtTPST protein showed low similarity (less than 15%) to any other AtSOT of Arabidopsis, we considered it to constitute a new group of the AtSOT protein family, group IX. In the present investigation, two members of Group IX showed distinct features compared with the other eight groups: they contained SOT_2 but not SOT_1 and their motif patterns were very different. Our results indicate that tyrosylprotein *SOT* genes should be classified into an independent group, Group IX.

Our phylogenetic analysis of the 56 BraSOT proteins and 22 AtSOT proteins separated the *B*. *rapa* SOT-domain proteins into nine groups (Fig 2). BraSOT members were not predominant in all groups. BraSOTs were more heavily represented than AtSOTs in seven groups, with BraSOT:AtSOT proportions as follows: group I (11:2), IV (5:1), V (12:2), VI (3:2), VII (14:3), VIII (5:3) and IX (2:1). In contrast, *BraSOTs* were less abundant than *AtSOTs* in groups II (1:4) and III (2:4). The two contrasting situations not only imply the occurrence of *BraSOT* gene triplication events along with recurrent genome duplication, but also reveal that *BraSOT* genes have undergone loss, fragmentation and dispersal after polyploidization during *Brassicaceae* evolution.

### Domain organization diversity and putative function analysis

Forty-eight BraSOT proteins contain only one SOT_1 domain and no other domains, while six BraSOT proteins have SOT_1 and SOT_2 domains and two proteins in Group IX have one SOT_3 domain. Transmembrane regions were only detected in six BraSOT proteins, all in groups VIII and IX, using the SMART sequence analysis tool [33], indicating that most BraSOT proteins are not associated with membranes. Bra034521 in Group I contains several pentatricopeptide repeat domains, which are possibly involved in RNA stabilization [36] or editing [37], suggesting that Bra034521 is involved in those functions in *B*. *rapa*. Bra017364 in Group V contains a translin domain. Translin-domain proteins are DNA-binding proteins that specifically recognize consensus sequences at breakpoint junctions in chromosomal translocations [38]. The role of Bra017364 in *B*. *rapa* is thus most likely related to chromosomal translocation. In Group VII, Bra027118 contains a coiled-coil domain. Such domains are involved in important biological functions, such as regulation of transcription factors, which suggests that Bra027118 is involved in the regulation of gene expression in *B*. *rapa*. The information presented here provides a useful reference for further study of the functions of these genes in *B*. *rapa*.

The motif patterns of the genes are relatively similar in each SOT group. All BraSOT proteins in groups I to VII possess conserved regions I and IV, which are involved in PAPS binding. These conserved regions are absent from groups VIII and IX, suggesting that group-VIII and group-IX BraSOT proteins have PAPS-binding regions that differ from those of other BraSOTs.

In summary, this investigation was the first systematic analysis of the SOT protein family in *B*. *rapa*. Our findings regarding gene and protein structure, gene distribution and protein classification should aid further investigations of the functions of *BraSOT* genes in *B*. *rapa* and will provide a useful reference for the study of *SOT* genes in other plants. Our data will also promote study of the evolution of polyploid genomes and the genetic improvement of *Brassica* oil and vegetable crops.

### The response of *B*. *rapa SOT* genes to abiotic stresses

It has been reported that *AtSOT12* gene expression was strongly induced by salt, osmotic stress and hormone treatments. The T-DNA knock-out mutant *sot12* exhibited hypersensitivity to NaCl and ABA during seed germination [20]. While ABA also has recently been reported to play crucial roles in responding to abiotic stresses, such as drought and salinity [39,40]. However, it is still unknown which *BraSOTs* is the important ABA receptor in response to abiotic stress in *B*. *rapa*. To investigate the possible roles of *BraSOT* genes in *B*. *rapa* growth and development, we analyzed the transcription of all 56 *BraSOT* genes by qRT-PCR (Fig 6). The *BraSOT* genes displayed different expression patterns, indicating that they may have different functions in *B*. *rapa* development. As shown in Fig 6, the expression of 5 *BraSOT* genes (*Bra034065*, *Bra017370*, *Bra009300*, *Bra027880*, *Bra015936*) was substantially up-regulated under PEG-6000, salinity and ABA treatment. The high expression levels of these genes indicate they have important roles in the plant response to abiotic stresses and the ABA signal transduction. However, some members of group V subfamily(*Bra017364* and *Bra026538*), grouped with *Bra017370*, were down-regulated in response to salt and drought stresses. This is similar to the *AtSOTs*. For example, the *AtSOT12* gene was clustered with and encoded a protein with high sequence similarity to *AtSOT11* and *AtSOT13*, but mutations in *AtSOT11* and *AtSOT13* did not cause a more sensitive phenotype to SA like *AtSOT12* mutation [20]. Our research suggests that some group-V *BraSOTs* are involved in plant responses to abiotic stresses, while others, even though they have similar sequences, seem to have no role in drought or salt stress responses. The functions of these genes require further study. Most *BraSOT* genes in group VIII and IX were down-regulated by drought, salinity and ABA treatment. Moreover, the structure of this two group genes were particularly different from other group. They have more introns (Fig 3). This may suggesting that these *BraSOTs* have disparate functional roles under abiotic stresses or involved in a negative-feedback regulatory mechanism. In Arabidopsis, *AtSOT12*, which is homologous to the *Bra017370*, is overexpressed to enhance abiotic tolerance. In our study, *Bra017370* expression level increased by drought, salinity and ABA. We thus speculated that overexpression of *Bra017370* could also promote abiotic resistance in *B*. *rapa*. ABA is produced rapidly in response to stress under drought and salinity conditions and then plays an important role in the regulation of the stress response [41]. Consequently, perhaps abiotic stress induced gene in this research (*Bra034065*, *Bra009300*, *Bra027880*, *Bra015936*) could also be overexpressed to enhance *B*.*rapa* abiotic tolerance. In conclusion, the results of the stress response experiments, combined with the analysis of the gene and protein structure (Figs 3 and 5), suggest that some *BraSOTs* respond to drought, salinity, and ABA treatments but have different mechanism. These *BraSOTs* may potentially be utilized for improving the tolerance of *B*. *rapa* to abiotic stresses.

## Supporting information

S1 Fig Before and after the abiotic treatment of B. rapa seedings. (TIF)

S1 Table qRT-PCR Primers for *BraSOTs* and control genes. (DOCX)

S2 Table Relative expression level of 56 *BraSOT* genes in different tissues and after drought, salinity, ABA treatment. (XLS)
