# Supplementary figures and images for: Genome-wide analysis of sulfotransferase genes and their responses to abiotic stresses in Chinese cabbage (Brassica rapa L.)
Source: PLoS One. 2019 Aug 19;14(8):e0221422. doi: 10.1371/journal.pone.0221422 (PMC6699706; doi:10.1371/journal.pone.0221422)

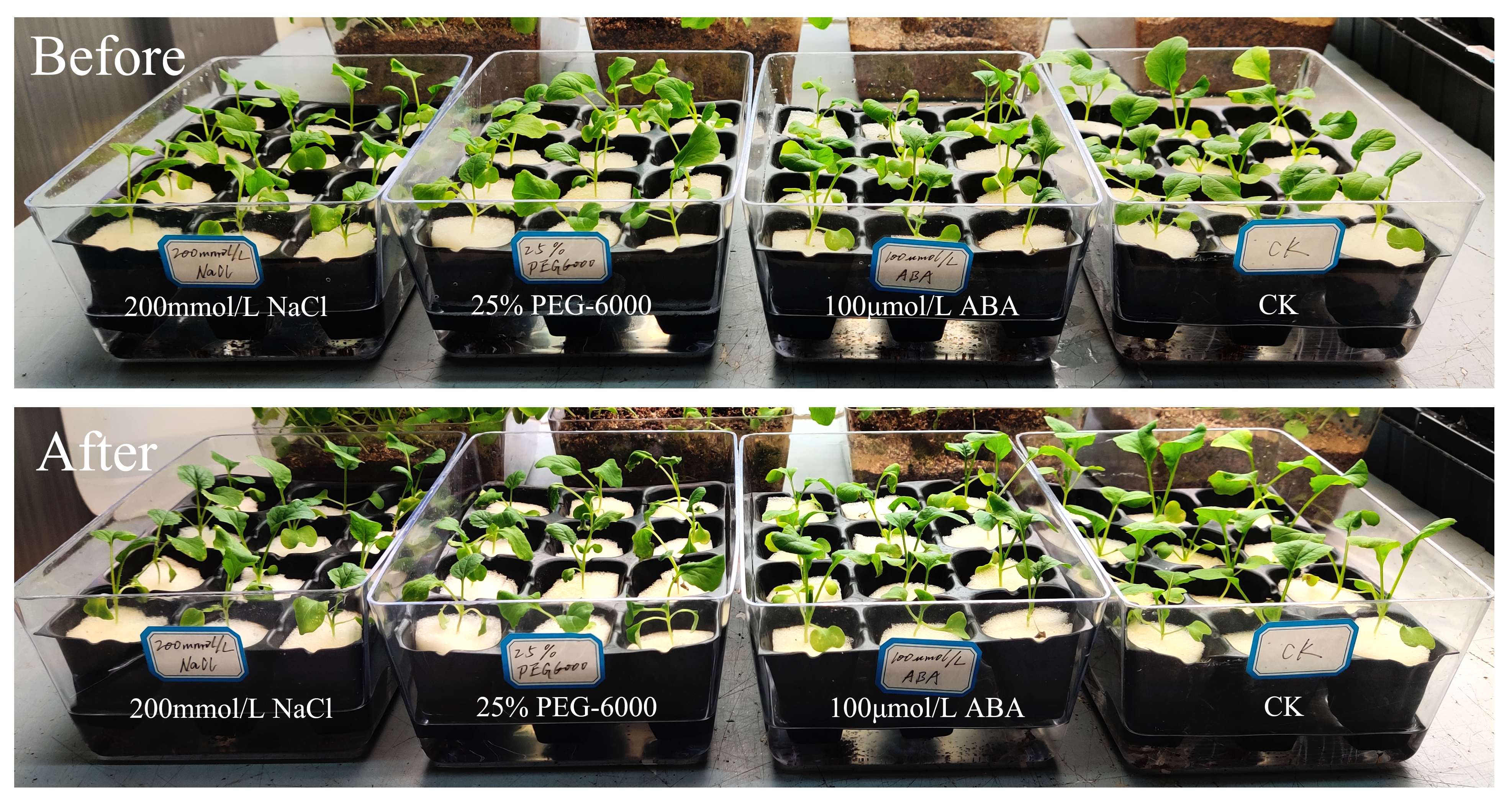

Supplement: S1 Fig — (TIF) [file pone.0221422.s001.tif]
