# Supplementary material for: Genome-wide analysis of sulfotransferase genes and their responses to abiotic stresses in Chinese cabbage (Brassica rapa L.)
Source: PLoS One. 2019 Aug 19;14(8):e0221422. doi: 10.1371/journal.pone.0221422 (PMC6699706; doi:10.1371/journal.pone.0221422)
Supplement: S1 Table — (DOCX) [file pone.0221422.s002.docx]

**Table S1.** qRT-PCR Primers for *BraSOTs* and control genes.

| **GeneID** | **Fprimer** | **Rprimer** |
| --- | --- | --- |
| AF111812 | GGAGCTGAGAGATTCCGTTG | GAACCACCACTGAGGACGAT |
| Bra026539 | GATCATGTGTTGGAGTATTGGC | TGTATCTCCAGTCTGCTTCTTG |
| Bra017365 | GCATCCTCTTCTAGCTACCAAT | CAGAGAATGATGCTGTAAGTGC |
| Bra017368 | TGGAAGGAGATTACTGCGTATC | GGAGCAAGTTTTCTCCCAAAAT |
| Bra017369 | TGCTCTCATTCACCAAAACAAG | TTTACAATCGACGTCTGGAGAT |
| Bra017371 | AGCTCTTCTCTTTGCTCTCATT | AACTTCCCTTTACAAAACGCTG |
| Bra017373 | GGCACACAAAAGCTATGTTACA | CTTGTTTTGGTGAATGAGAGCA |
| Bra017364 | GATGCTTTCTCTCAATACGCTG | TGTTCCCTCACTGCTTCTAAAT |
| Bra017370 | TTCTTCTTCTTCTGGTGAGCAT | ATGTGATATGTGCGTGTTCATG |
| Bra017372 | AACCGCTGACTATCCTAT | GCATACCAATACTCCAACA |
| Bra026535 | CTTACAAGGACTCATGGAGTGT | AAGAAACTGGAAACTTGTGTCG |
| Bra026538 | AGAAGTGAGAGGGATTGTGAAG | TTGGCAACTTCCCTTCTCTATT |
| Bra005879 | CCACCAGGAGGCACATTATC | CCCTTTCCAAAGGTTTTTGGTGG |
| Bra005876 | AGAAGAGAGCGGAGCTGTGG | CACTCCGTTAGACGTTTTCCCT |
| Bra005878 | ATGAACTTCCAGCGACCGTG | TGGTACTTGCAGAGGTTGCC |
| Bra009164 | CGCGTTGGTATTTCAGGAGC | CTCCAGTAACTGAGGGCGTG |
| Bra004046 | CTCACATGCCATTCCACACG | CCAAAAGAGCCAACACCTTCG |
| Bra016726 | AGGAAATGAAGTCAGAGCCCG | TCTTGTCCACAGATCCGCCT |
| Bra004349 | TTCACTGAGGAAGAGCACGG | TTGGAGTCACCGACTTCTCC |
| Bra036654 | AGCATTCTGCAACGGAGCTA | AGGCTCGGCTTTCATTTCCT |
| Bra034520 | GGTATCAGAACCAGGTTGACAAG | TGCCATACACACCTCCCAAT |
| Bra034521 | ACCTCCCGTTGTTATCTTCCTT | TGCGCTGAAACTAGGGACTT |
| Bra027666 | CGTACCCCAAATCAGGCACT | AGGACTTGAGCTTTCGTGGT |
| Bra017006 | GGAAAGGTGTGGTTGGAGATTG | TCCAAGCCAGATTCACTCCAT |
| Bra026450 | AAGAGTACCTTGGTGGAAACCTC | ACCGCATTTGGGGAAGGATG |
| Bra041015 | CGGAGAACCCTTCTTCTGGTG | AAGTCCGGTTTGTGGGTGTC |
| Bra010899 | TCGTTCCCTAAATCCGGCAC | TGGTTTCGAGGTTTCGGTGT |
| Bra034065 | TGCAAAGTCGTTTACATGTGCAG | AAACGGTCCCAACGAGTAAACC |
| Bra034066 | TTGGCATTTCAGGAGCAAGC | CCCGAAGAAGCAAACCCCTT |
| Bra026540 | TCCCTCATGACCTTGTCCCA | ACTTTGTGGTGGCTTCCCTC |
| Bra027963 | TGAGCTACTGGAAAGGGAGC | ACCGAGTCTCTTGACTTGAAGG |
| Bra036913 | TGAGGAGCCACTTATTCAGGTG | AGTTCAAGATCTCCTCCGCTG |
| Bra005921 | TCTCCTCGTGGCATTTCAGC | AGCATGTGTTCCCAAAACGG |
| Bra009300 | AGAGCTTGAGGACACCAGAGA | GAAGCTGCACAGATCCGAGA |
| Bra028711 | AGGGTTTGGTCCGTTTTGGG | ACGATTGCCTTCGTAACACC |
| Bra003818 | CCGACACTACCGTTCCAAAC | CCAATGACCACCGTACCTGA |
| Bra003726 | TATGCCCCATGAGTTATTACCC | TTGAAAGAAATGCCACAAGGAG |
| Bra003817 | GACGATTCCACGAACCCTCT | GGTCTCTCCAGATATAAACCCGAT |
| Bra027623 | GTGAGTGCTGATCCTTTGCC | CGCGCGGTTTACATTAGGAG |
| Bra003819 | TTATCTCTGCACGGTCCTTATC | ATGAACTCAGCCAATCTCTTCA |
| Bra027118 | GACGAGGCCAAGATAGCGTC | ACCACAGTACCCGATGAACG |
| Bra027880 | TGGAGTCAGCAATCGACACG | ACGTAGCGATCAGGTCTTGG |
| Bra027117 | ATGCAACCCTACCGAAGCAC | TCAGCGGAGCTTTTGGTCTC |
| Bra031476 | TCTTTTCTCGACTCATATCCCG | TTGGGTCTCTCCAGATGTAAAC |
| Bra015938 | TGGAATCAGAGCCCTTAACCG | TCGGTCTTGTCTTGGTTCGG |
| Bra015936 | GACAGGAGGACCAAACCAGG | CACCAGCTACCACCGTACTC |
| Bra025668 | GCTCAAGGCACTAAACTGCG | TGAGGGTTGCGTTTGAGGAG |
| Bra008132 | CGAACTCGAACTCGAACTCTC | AGCCTTTGCTCTTTGGGAGG |
| Bra015935 | TCTCAAGAGGGTCAGCAGCTA | CAGCTCTCATCGTCTCGTACC |
| Bra036094 | CTTGGTACTGCAGGCAGATG | AACCTCACCGTCCGTTTTGT |
| Bra012895 | CTCTGTTCTTGTCTGTGTGTCG | GTTTCGAACCAACCGCTTCC |
| Bra011532 | GCGCATTGTGGTTTGTTACATCT | TTGCCGACTCTTTAACTCACG |
| Bra013096 | TTCAGGCTGAGATACTGGCG | ATATGTTCTCACGGTGCGGT |
| Bra015232 | AGACTGACTGTCTCTTTTTATCGC | TGTCGTCTGAACCTCGTTCC |
| Bra018664 | ACAGACGCGTACAAGAGTTCC | CTCTGAGCCAGTTCCATTGC |
| Bra030710 | CGTTTCAACAGTAAGTCCCTCT | AGCATCAATGCCACTGTCCA |
